# Mortality and Its Associated Factors among Hospitalized Heart Failure Patients : The Case of South West Ethiopia

**DOI:** 10.1155/2021/5951040

**Published:** 2021-08-24

**Authors:** Meiraf Daniel Meshesha, Robel Hussen Kabthymer, Mohammed Mecha Abafogi

**Affiliations:** ^1^Department of Internal Medicine, College of Health Science and Medicine, Dilla University, Dilla, Ethiopia; ^2^Institute of Health, Jimma University, Jimma, Ethiopia; ^3^School of Public Health, College of Health Science and Medicine, Dilla University, Dilla, Ethiopia

## Abstract

**Background:**

Hospital case fatality among those with heart failure in Africa ranges from 9% to 12.5%. An integrated approach to identify those who are at high risk and implementing specific treatment strategies is of great importance for a better outcome.

**Objective:**

The aim of this study is to assess the mortality rate and its associated factors among hospitalized heart failure patients at the Jimma University Medical Center (JUMC), south west Ethiopia.

**Method:**

A hospital-based retrospective cross-sectional study design was conducted among 252 patients admitted with heart failure during the study period who were sampled and enrolled in to the study. A simple random sampling technique was used to select the study participants by using their medical registration number as the sampling frame. Data were collected using a pretested questionnaire. The collected data were entered into EpiData software and exported to SPSS version 20 for cleaning and analysis. A binary logistic regression model was used. Adjusted and crude odds ratio with 95% CI were used. A *P* value less than 0.05 was used to declare statistical significance.

**Results:**

The prevalence of in-hospital mortality was found to be 21.29%. Cardiogenic shock AOR: 0.016 (95% CI: 0.001–0.267), complication at admission AOR: 5.25 (95% CI: 1.28–21.6), and ejection fraction (<30) AOR: 0.112 (95% CI: 0.022–0.562) were found to be significantly associated factors.

**Conclusion:**

The in-hospital mortality rate among admitted heart failure patients is unacceptably high. Due emphasis should be given on the identified associated factors to reduce the mortality.

## 1. Introduction

Worldwide, the burden of cardiovascular disease (CVD) is increasing. This is mainly attributed to the sharp rise in the incidence of CVD among developing countries [1]. Heart failure is among these CVD contributing to 3–7% of the hospital admission rate in Africa [2].

HF is a clinical syndrome characterized by its typical symptoms (e.g., shortness of breath, ankle swelling, and fatigue) which may be accompanied by signs (e.g., raised JVP, pulmonary crackles, and peripheral edema). A structural and/or functional cardiac abnormality can result in a reduced cardiac output and/or elevated intracardiac pressures at rest or during stress [3].

Hospitalization for heart failure can be due to a sudden or gradual deterioration of a stable HF. According to [3], whichever the scenario is, being hospitalized for heart failure poses a significant risk for the patient [4]. Hospital case fatality among those with heart failure in Africa ranges from 9% to 12.5% [5].

Regardless, the integrated approach to identify those which are at a higher risk and implementation of specific treatment strategies is of great importance as it can lead towards a better outcome. The goals of treatment include improving the hemodynamic status, organ perfusion, and oxygenation urgently, limiting further organ damage, treating acute precipitants, preventing the development of complications, and initiation of the long-term, evidence-based disease-modifying agents that can alter the natural course of the illness. In 2015, an estimated 17.7 million deaths occurred due to cardiovascular diseases, accounting for more than 30% of the overall total of 56 million deaths [6]. Heart failure (HF) is among these cardiovascular diseases, with a major public health impact worldwide. The prevalence of HF is approximately 1-2% of the adult population in developed countries, rising to ≥10% among people >70 years of age. [3] Approximately 5 million patients in America have heart failure, and over 550000 patients are diagnosed with HF for the first time each year [7].

HF is also one of the leading causes of hospital admission in the world. In Africa, previous studies have revealed that heart failure accounts for over 30% of hospital admission in specialized cardiovascular units and 3%–7% in general internal medicine wards which are similar to the rate in developed countries of Western Europe and America [5]. In Zimbabwe, patients with heart failure contribute to about 6% of hospital admission; this was also associated with a significant increment in the proportion of death resulting from heart failure [8].

Compared to studies from other parts of the world, heart failure in Africa tends to occur at a much younger age with most cases recorded around the 5th and 6th decade [5].

Admission for heart failure is a high-risk event for patients. A study from USA revealed the in-hospital mortality for hospitalized HF patients of 4% [4]. A rate of 6.7% was reported from Europe [9]. A report from THESUS-HF conducted in 9 sub-Saharan African countries found an in-hospital mortality rate of 4.2%, similar to registries in US and Europe despite differences in the cause of HF and age at presentation [10]. Hospital case fatality among those with heart failure in Africa ranges from 9% to 12.5%. This consistent death rate ranks heart failure among the major causes of death of cardiovascular origin in Africa [5].

In Ethiopia, adequate studies were not conducted to describe the rates of mortality in hospitalized patients with heart failure. Furthermore, important factors associated with in-hospital mortality have not been addressed in this specific population. Since major genetic and environmental influences affect the course of any disease process, including heart failure, a study specifically designed to a subgroup of population can reveal major differences in this aspect. This study aims to describe the in-hospital mortality rate and the important associated factors among heart failure patients residing in the catchment area of the Jimma University Medical Centre, Jimma, Ethiopia, with an effort to explore this relatively uncovered research area.

## 2. Materials and Methods

### 2.1. Study Area and Period

The study was conducted at the Jimma University Medical Centre, one of the teaching hospitals in Ethiopia located in the Oromia Region, Jimma Zone, Jimma town. Jimma town is located at about 346 km, south west of Addis Ababa. It provides services for approximately 9000 inpatient and 80,000 outpatient attendants in a year who come to the hospital from the catchment population of about 15 million people residing within the catchments area of 250 km radius. Patients who need hospitalization for heart failure are directly admitted to general medical wards and are managed with a team of clinicians which include nurses, medical interns, residents, internists, and cardiologists.

The study was conducted from February 1, 2018–May 31, 2018.

The study was registered on http://www.researchregistry.org with a unique identification number of research registry 7018. The study was reported in line with 2019 STROCSS criteria (http://www.strocssguideline.com).

### 2.2. Study Design

A hospital-based, retrospective, cross-sectional study was conducted.

### 2.3. Source Population

All cases of heart failure patients aged ≥14 years admitted to the Jimma University Medical Centre general medical wards from September 11, 2016, to September 10, 2017, were considered.

### 2.4. Study Population

All cases of randomly selected eligible heart failure patients aged ≥14 years admitted to the Jimma University Medical Centre general medical wards from September 11, 2016, to September 10, 2017, were considered.

### 2.5. Inclusion Criteria

(i) All cases of admitted heart failure patients aged ≥14 treated in the Jimma University Medical Centre from September 11, 2016, to September 10, 2017, were included in the study.

### 2.6. Exclusion Criteria

- Those medical records where the chart was lost and/or there were incomplete data on outcome variable were excluded from the study
- Records of those who were readmitted within the study period were excluded

### 2.7. Sampling Size

(i) Sample size was calculated using a single population proportion formula:
  (1) $$\begin{array}{c} n_{0}={\left(\frac{Z_{1-\alpha }}{\delta }\right)}^{2}p\left(1-p\right). \end{array}$$

The sample size was calculated based on the assumption of the margin of error of 5%, with 95% confidence interval. Since there is no prior experience regarding mortality of patients hospitalized with heart failure in the setup, sample proportion was taken as 50%. This would give a sample size of **385**. This was again corrected for a population size of *N* < 10000, using the following formula:

(2) $$\begin{array}{c} n=\frac{n_{0}}{1+n_{0}/N},\\ n_{0}=385N=566,\\ n=229. \end{array}$$

Finally, an additional 10% was added for the nonretrieval rate 229 + 23 = **252**.

### 2.8. Sampling Procedure

To select study participants randomly, openepi software version 2.3 was used to generate random numbers. First, medical record number was retrieved from the patient registry in the wards. Medical record number from smallest to the highest was entered to software to select the calculated sample size. Thereafter, using the corresponding medical record number, a random sample was selected and patients' card was retrieved from the record room based on the medical record number.

### 2.9. Data Collection Method

Data were collected using a pretested data collection tool. A structured and pretested data collection tool was used to collect the data from medical records. They were collected from registries of patient intake forms, and patients' cards were retrieved from the card room using the medical record number. Six medical interns were recruited to collect the data from the patient medical record. Before actual data collection, the data collection tool was pretested. One supervisor with a principal investigator followed the data collection closely.

### 2.10. Operational Definition

- Anemia: a hemoglobin level of <13 g/dl and <12 g/dl in men and women, respectively
- Cardiogenic shock: heart failure accompanied by low blood pressure (SBP <90 mmHg) and a diagnosis of cardiogenic shock made by a physician
- Heart failure: a clinical syndrome with a physician diagnoses of heart failure
- Hypertensive heart failure: high blood pressure (>180/100 mmHg) accompanied by symptoms of HF (dyspnea and tachycardia) and radiological findings of pulmonary congestion or edema and with preserved left ventricular (LV) function
- New-onset (“de novo”) heart failure: a sudden or gradual development of sign and symptoms of HF for the first time
- Prolonged QTc: a QTc value >440 ms and >460 ms in males and females, respectively, or more than 500 ms if there is ventricular depolarization abnormality
- Pulmonary edema: a physician diagnosis of pulmonary edema presumed to be of cardiogenic cause
- Renal impairment: a serum creatinine of >1.2 mg/dl

### 2.11. Data Quality Control

To assure data quality, the data were pretested on 5% of total sample size of patients admitted with HF in the Jimma University Medical Centre. After the pretest, the necessary amendment of tool was used for the final data collection. Then, the data collection tool was corrected and data collectors were made aware of the data collection. Intensive two-day training was given for six data collectors on how to extract the data from the patient registry. The daily collected data were checked by the supervisor and principal investigator for completeness and consistencies.

Double data entry to EpiData software by two independent data clerks was carried out. Then, the two datasets were validated in the software for consistency and mismatches were checked with cross checking with the questionnaire until the two datasets match perfectly.

### 2.12. Data Processing and Analysis

After entering and checking the data with EpiData software Version 3.02, the data were exported to SPSS version 24 for cleaning and analysis. Percentage (number) was used for categorical variables.

Independently associated factors of mortality were identified using the binary logistic regression model. Adjusted and crude odds ratio with 95% CI were used. *P* value less than 0.05 was used to declare statistical significance.

## 3. Results

The overall prevalence of mortality among hospitalized heart failure patients in this study was found to be 21.29%, and a majority (79.91%) of them was discharged as improved.

**Figure 1:**
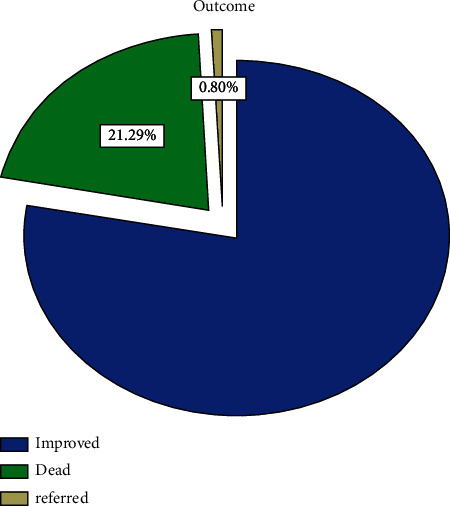
Pie chart showing the treatment outcome of hospitalized heart failure patients at the JUMC from September 11, 2016, to September 10, 2017.

As shown in Figure 1, mortality rate among admitted heart failure patients is 21.29%, whereas a majority of them, i.e., 79.91%, were discharged improved. More than half (54.8%) of the participants were females, and a big share (30.2%) of participants were found to be in the 45–60 age category (Table 1).

At admission, a majority (71%) of the patients had a systolic blood pressure in the range of 90–140; similarly, a majority (71.4%) of the participants had a diastolic BP in the range of 60–90. Most (64.2%) of the patients had a pulse rate in the range of 100–120 mmHg, and 191(75.8%) had respiratory rate >24. Only 3.2% of the patients had cardiogenic shock at admission, and 16.7% of the patients had pulmonary edema (Table 2).

Regarding laboratory findings, 149 (60.3%) of the patients had a hemoglobin level of less than or equal to 12 and a majority 179 (71.9%) had a creatinine >1.2, whereas 90.4% of the patients had a BUN level of greater than 20 and 60.4% of them were found to have hyponatremia (Table 3).

Regarding ECG and echocardiographic findings, 54.6% were found to have some form of arrhythmia at admission, and 44.2% of the participants had long QTc. An ejection fraction in the range of 30–50% was found in 37.6% of the patients (Table 4).

Regarding the previous history and complication-related factors, 145 (57.5%) of the patients did not have a previous diagnosis of heart failure. Nearly sixty percent of the patients were without a previous history of hospitalization for HF. Close to half (53.2%) of the patients were not found to have any comorbidity at admission. One hundred and forty (55.6%) of the patients did not have any complication at admission, whereas 94% of the patients developed some form of complication after admission (Table 5).

Regarding treatment-related factors, almost all (98%) of the patients have not taken parenteral inotropes, but 143 (56.7%) patients have taken beta-blockers and nearly half (51.6%) of the patients have taken ACEI. Sixty-three (25%) patients were on ventilatory support. Length of stay for more than half (62.2%) of the patients was 7–30 days (Table 6).

### 3.1. Bivariate and Multivariate Analysis

On bivariate analysis, sex, systolic Bp, diastolic Bp, pulse rate, respiratory rate, cardiogenic shock, pulmonary edema, arrhythmia, long QTc, complication at admission, complication after admission, use of B-blocker, ACEI, and serum sodium were found to be candidate for multivariable analysis with *P* value <0.25.

On multivariable analysis, three significantly associated independent variables were found. Patients admitted with complication are 5.25 times more likely to die as compared to those patients admitted without complication. Patients without cardiogenic shock are 98.4% less likely to die as compared to those patients with cardiogenic shock. Patients with an ejection fraction of 30–50% were 88.8% less likely to die as compared to those patients with an ejection fraction of ≤30 (Table 7).

## 4. Discussion

This study has tried to assess the in-hospital mortality rate among patients admitted with acute heart failure and its associated factors. In this study, the in-hospital mortality rate was found to be 21.29%. This was much higher than that found in studies carried out in other countries. A study from Brazil found an in-hospital mortality rate of 11.2% [11]. Similarly, a prospective study carried out in India found an in-hospital mortality rate of 11%. [12].

From reports in Africa, the Sub-Saharan Africa Survey of Heart Failure (THESUS–HF) which was conducted in 1006 patients with AHF admitted to 12 university hospitals in 9 countries had the lowest rates of in-hospital mortality rate of 4.2% [10], while a study from Cameroon shows an in-hospital mortality rate of 9% and a Nigerian study shows a 15.7% mortality rate [13, 14].

The higher mortality rate which was found from our study can be explained by the fact that there is excessive patient load due to a very large catchment area of the hospital together with lack of adequate infrastructures including advanced care services. Delayed referral to the center from primary-care centers might also contribute to the scenario. Moreover, some differences in the characteristics of our patient might also be a contributing factor.

Although there are reports which indicate the NYHA functional class should not solely be dependent in assessing the severity, prognosis, and guiding management [15], some studies have found that the NYHA classification significantly predicted mortality in hospitalized patients [11, 12]. In our study, the NYHA class was not found to be significantly associated with mortality. Yet, most of our patients were at NYHA class IV (44%) or class III (42%) which is significantly higher than that in a report from sub-Saharan Africa where a majority (42.9%) of patients were at class II and 30.6% of them were at class III [10].

Previous hospitalization in a heart failure patient was found to be a significant predictor of in-hospital mortality in a study from Brazil [11]. Regardless, our study revealed that about 60% of the study population has not had a previous history of hospital admission and it was not found to be significantly associated with mortality.

As to the cause of heart failure, unlike most reports from Africa [10, 14], where hypertension dominates, the most common underlying etiology in our study was ischemic heart disease (41.7%) followed by rheumatic heart disease (18.7%) and idiopathic dilated cardiomyopathy (14.7%).

In our study, the presence of any complications at admission was strongly associated with mortality, as those patients admitted with complication were 5.25 times more likely to die as compared to those patients admitted without complication. This finding was also evidenced from a study conducted in Brazil, which showed that the nonsurvivor group had more complications during hospitalization, such as pulmonary thromboembolism, need for dialysis, and respiratory infection [11].

The presence of cardiogenic shock was another factor found to be strongly associated with mortality in our study. Patients without cardiogenic shock were 98.4% less likely to die as compared to those patients with cardiogenic shock. This finding goes in line with findings from other studies such as a study from Italy on in-patient outcome of AHF which reported the highest mortality rate (23.8%) in patients with cardiogenic shock. A strong association was also found between in-hospital mortality and SBP [16]. The Euro Heart Failure Survey II also found that, among clinical groups, in-hospital mortality was extremely high in cardiogenic shock patients (39.6%) [9].

Furthermore, LVEF was also found to be a strongly associated factor with mortality in our study. It was revealed that patients with a higher ejection fraction of 30–50% were 88.8% less likely to die as compared to those patients with lower ejection fraction, ≤30. Other studies also noted this similar effect of lower LVEF on mortality. As an example, a report from India showed that the severity of systolic dysfunction was among the strong predictors of in-hospital mortality [12]. Another evidence comes from Nigeria in 2007 on hospitalized AHF patients in which all the reported deaths had an LVEF ≤40. [17]. But, these results should be interpreted cautiously since LVEF has many limitations in assessing left ventricular function including inter-and intraobserver variability, its dependence on preload and after load, and image quality. Moreover, pathophysiologic classification of heart failure may also be inaccurate [18–20].

### 4.1. Limitation of the Study

Difficulty of ascertaining the reliability of recorded secondary data and cross-sectional nature of the study design which fails to show clear temporality of the factors associated with in-hospital mortality are some of the limitations of the study.

## 5. Conclusions

The findings of our study revealed an unacceptably high in-hospital mortality rate among patients hospitalized with heart failure. Moreover, the presence of complications at admission, cardiogenic shock, and a lower LVEF were found to have a statistically significant association with in-hospital mortality among patients admitted with heart failure.

## Figures and Tables

**Table 1:** Sociodemographic characteristics of hospitalized heart failure patients at the JUMC, Jimma, Ethiopia, from September 11, 2016, to September 10, 2017.

Variables	Category	Frequency	Percentage
Sex	Male	114	45.2
	Female	138	54.8

Age	15–30	54	21.4
	30–45	58	23.0
	45–60	76	30.2
	≥60	64	25.4

**Table 2:** Clinical presentation-related characteristics of hospitalized heart failure patients at the JUMC, Jimma, Ethiopia, from September 11, 2016, to September 10, 2017.

Variables	Category	Frequency	Percentage
Systolic BP	≥140	64	26.1
	90–140	174	71.0
	<90	7	2.9

Diastolic BP	≥90	58	23.7
	60–90	175	71.4
	<60	12	4.9

Pulse rate	≥120	54	35.8
	100–120	97	64.2

Respiratory rate	>24	191	75.8
	14–24	61	24.2

Cardiogenic shock	Yes	8	3.2
	No	244	96.8

Pulmonary edema	Yes	42	16.7
	No	210	83.3

**Table 3:** Laboratory result-related characteristics among hospitalized heart failure patients at the JUMC, Jimma, Ethiopia, from September 11, 2016, to September 10, 2017.

Variables	Category	Frequency	Percentage
Hemoglobin	>12	98	39.7
	≤12	149	60.3

Creatinine	>1.2	179	71.9
	≤1.2	70	28.1

BUN	>20	222	90.6
	≤20	23	9.4

Sodium	Hyponatremia	67	60.4
	Normal	44	39.6

**Table 4:** ECG and echocardiographic findings of hospitalized heart failure patients at the JUMC, Jimma, Ethiopia, from September 11, 2016, to September 10, 2017.

Variables	Category	Frequency	Percentage
Arrhythmia	Yes	95	54.6
	No	79	45.4

Long QTc	Yes	76	44.2
	No	96	55.8

Ejection fraction	<30	60	34.7
	30–50	65	37.6
	>50	48	27.7

**Table 5:** Past history-and complication-related characteristics of hospitalized heart failure patients at the JUMC, Jimma, Ethiopia, from September 11, 2016, to September 10, 2017.

Variables	Category	Frequency	Percentage
Previous HF	Yes	107	42.5
	No	145	57.5

History of hospitalization for HF	None	145	60.4
	1 hospitalization	39	16.3
	>1 hospitalization	24	10.0
	Not mentioned	32	13.3

Comorbidity	Yes	118	46.8
	No	134	53.2

Complication at admission	Yes	112	44.4
	No	140	55.6

Complication after admission	Yes	237	94.0
	No	15	6.0

**Table 6:** Treatment-related characteristics of hospitalized heart failure patients at the JUMC, Jimma, Ethiopia, from September 11, 2016, to September 10, 2017.

Variables	Category	Frequency	Percentage
Pare inotropes	Yes	5	2.0
	No	247	98.0

Beta-blocker	Yes	143	56.7
	No	109	43.3

ACEI	Yes	130	51.6
	No	122	48.4

Ventilation support	Yes	63	25.0
	No	189	75.0

Length of stay	<7	76	30.5
	7–30	155	62.2
	≥30	18	7.2

**Table 7:** Multivariable model showing significantly associated variables.

Variables	Category	Frequency	AOR	95% CI	*P* value
Complication at admission	Yes	112	5.25	1.28–21.6	0.021^*∗*^
	No	140	1		

Cardiogenic shock	Yes	8	1	0.001–0.267	0.004^*∗∗*^
	No	244	0.016		

Ejection fraction	≤30	60	1		
	30–50	65	0.112	0.022–0.562	0.008^*∗∗*^
	≥50	48	1.365	0.115–16.16	0.805

^*∗∗*^Variables with a *P* value of <0.01, ^*∗*^variables with a *P* value of <0.05.

## Data Availability

The data used to support the findings of this study are available from the corresponding author upon request.

## Abbreviations

ACE: Angiotensin-converting enzyme
ACS: Acute coronary syndrome
AF: Atrial fibrillation
ACEI: Angiotensin-converting enzyme inhibitor
ADHF: Acute decompensated heart failure
AHF: Acute heart failure
AKI: Acute kidney injury
ALP: Alkaline phosphatase
ALT: Alanine transaminase
AMI: Acute myocardial infarction
AOR: Adjusted odds ratio
AST: Aspartate transaminase
AV: Atrioventricular
BNP: Brain natriuretic peptide
BP: Blood pressure
BUN: Blood urea nitrogen
CI: Confidence interval
CKD: Chronic kidney injury
COPD: Chronic obstructive pulmonary disease
Cr: Creatinine
CVD: Cardiovascular diseases
DCM: Dilated cardiomyopathy
EF: Ejection fraction
eGFR: Estimated glomerular filtration rate
HF: Heart failure
HHD: Hypertensive heart disease
IHD: Ischemic heart disease
JUMC: Jimma University Medical Centre
LVEF: Left ventricular ejection fraction
LVH: Left ventricular hypertrophy
NSTEMI: Non-ST-elevation myocardial infarction
NYHA: New York Heart Association
SBP: Systolic blood pressure
SPSS: Statistical Package for Social Science
STEMI: ST-elevation myocardial infarction
TIA: Transient ischemic attack.
